# Diagnostic value of ultrasound combined with MRI in cholecystolithiasis: A protocol for systematic review and meta analysis

**DOI:** 10.1097/MD.0000000000025896

**Published:** 2021-05-14

**Authors:** Mingfang Yang, Yu Shi

**Affiliations:** Beibei Traditional Chinese Medical Hospital, Chongqing, China.

**Keywords:** cholecystolithiasis, diagnostic test, MRI, systematic review, ultrasound

## Abstract

**Background:**

Early diagnosis of cholecystolithiasis is significant for prevention of further development of situation. Ultrasound is the best choice for the diagnosis of cholecystolithiasis with a sensitivity of >95% and specificity of practically 100%. However, ultrasound is not perfect for it is not so clear sometimes. So, MRI is needed to assist the diagnosing of cholecystolithiasis. Some studies have been conducted to investigate the diagnostic value of ultrasound combined with MRI in cholecystolithiasis, however, the evidence was not enough.

**Methods:**

We will search the following sources for the identification of trials: The Cochrane Library, PubMed, EMBASE, Chinese Biomedical Literature Database (CBM), Chinese National Knowledge Infrastructure Database (CNKI), Chinese Science and Technique Journals Database (VIP), and the Wanfang Database. The searches were limited to articles published before 1st, April, 2021, and the language were limited to Chinese and English. Statistical analyses will be conducted with Sata 14.0 software and the evaluation of the quality of the included studies will be performed by the Quality Assessment of Diagnostic Accuracy Studies (QUADAS-2).

**Results:**

This study will provide a rational synthesis of current evidences for MRI combined with ultrasound for cholecystolithiasis.

**Conclusion:**

The conclusion of this study will provide evidence for the diagnostic value of MRI combined with ultrasound for cholecystolithiasis.

**Ethics and dissemination:**

This protocol will not evaluate individual patient information or affect patient rights and therefore does not require ethical approval. Results from this review will be disseminated through peer-reviewed journals and conference reports.

**PROSPERO registration number:**

INPLASY202130003

## 1 Introduction

Cholecystolithiasis, also known as gallstone disease, is a common disease particularly among middle-aged women, as point estimates indicate a prevalence of 5–22%.^[1,2]^ There are several ways of diagnosis for cholecystolithiasis, of which ultrasound is the best with sensitivity of >95% and specificity of practically 100%.^[3]^ Though ultrasound sounds best it is not so clear sometimes. So, MRI is needed to assist the diagnosing of cholecystolithiasis. Some studies have been conducted to investigate the diagnostic value of ultrasound combined with MRI in cholecystolithiasis, however, the evidence was not enough. Therefore, we performed a meta-analysis to summarize these studies and more fully assess the diagnostic value of MRI combined with ultrasound for diagnosing cholecystolithiasis.

## 2 Method

### 2.1 Ethic committee or institutional review board

This protocol will not evaluate individual patient information or affect patient rights and therefore does not require ethical approval.

### 2.2 Study registration

This systematic review protocol was registered with INPLASY 2020 (registration number: INPLASY202130003).

### 2.3 Literature search

We will search the following sources for the identification of trials: The Cochrane Library, PubMed, EMBASE, Chinese Biomedical Literature Database (CBM), Chinese National Knowledge Infrastructure Database (CNKI), Chinese Science and Technique Journals Database (VIP), and the Wanfang Database. The search strategy will be as follows:(“ ultrasound imaging” OR “ultrasonic tomograph” OR “ultrasonography”) AND (“MRI” OR “magnetic resonance imaging”) AND (“Cholecystolithiasis”).

### 2.4 Study selection

Two reviewers will independently select the eligible literature. A third reviewer will be set for the solving of disagreement between two reviewers through discussion. There were no restrictions to patient age, race or nationality. The entire text of the journal articles that meet the eligibility criteria of this review will be acquired and will be reviewed by two authors. And the exclusion criteria will be as follows:

1. duplicate study,
2. do not obtain full text,
3. case reports, reviews, and seminar articles.

### 2.5 Data extraction

The following data will be extracted from the selected studies by two independent reviewers using a standard data extraction sheet: the first author's name, publishing data, sample size, blinding method, ultrasound frequency, field of MRI, research type, diagnostic values including sensitivity, specificity, true positive (TP), false positive(FP), false negative(FN), true negative(TN), and area under curve (AUC).

### 2.6 Quality assessment

QUADAS-2 (Quality Assessment of Diagnostic Accuracy Studies 2) tool will be used to assess bias and evaluate studies on diagnostic accuracy.^[4]^ Each item in the QUADAS-2 will be marked with “yes”, “no” or “unclear”, representing low risk, high risk and unclear, respectively.

### 2.7 Statistical analysis

The initial steps of data synthesis will include calculation of statistics including specificity and sensitivity with corresponding 95% CI and positive and negative likelihood ratios from diagnostic 2 × 2 tables of primary studies. Spearman's correlation analysis will be performed to examine a threshold effect. An I^2^ value more than 75% indicates significant heterogeneity and if encountered the data will not be combined. A bivariate random-effects regression method will be used to estimate specificity and sensitivity. Some modifiers will be applied for further evaluating of meta-regression and subgroup analyses. Deeks’ funnel plot will be developed to evaluate publication bias. All statistical analyses will be performed using Stata 14.0 software.

## 3 Discussion

This is a systematic review conducted to investigate the diagnostic value of ultrasound combined with MRI in cholecystolithiasis. It contains four sections such as identification, study inclusion, data extraction, and data synthesis. This review will aid doctors in the decision-making process for diagnosing cholecystolithiasis for patients.

## Acknowledgments

None declared.

## Author contributions

**Funding acquisition:** Yu Shi.

**Resources:** Mingfang Yang.

**Software:** Mingfang Yang.

**Writing – review & editing:** Yu Shi.
